# BRCA1 185delAG Mutation Enhances Interleukin-1*β* Expression in Ovarian Surface Epithelial Cells

**DOI:** 10.1155/2015/652017

**Published:** 2015-08-19

**Authors:** Kamisha T. Woolery, Mai Mohamed, Rebecca J. Linger, Kimberly P. Dobrinski, Jesse Roman, Patricia A. Kruk

**Affiliations:** ^1^Department of Pathology & Cell Biology, University of South Florida, Tampa, FL 33612, USA; ^2^Department of Medicine, University of Louisville, Louisville, KY 40202, USA; ^3^Department of Obstetrics & Gynecology, University of South Florida, Tampa, FL 33612, USA; ^4^H. Lee Moffitt Cancer Center, Tampa, FL 33612, USA

## Abstract

Familial history remains the strongest risk factor for developing ovarian cancer (OC) and is associated with germline BRCA1 mutations, such as the 185delAG founder mutation. We sought to determine whether normal human ovarian surface epithelial (OSE) cells expressing the BRCA1 185delAG mutant, BRAT, could promote an inflammatory phenotype by investigating its impact on expression of the proinflammatory cytokine, Interleukin-1*β* (IL-1*β*). Cultured OSE cells with and without BRAT were analyzed for differential target gene expression by real-time PCR, western blot, ELISA, luciferase reporter, and siRNA assays. We found that BRAT cells expressed increased cellular and secreted levels of active IL-1*β*. BRAT-expressing OSE cells exhibited 3-fold enhanced IL-1*β* mRNA expression, transcriptionally regulated, in part, through CREB sites within the (−1800) to (−900) region of its promoter. In addition to transcriptional regulation, BRAT-mediated IL-1*β* expression appears dualistic through enhanced inflammasome-mediated caspase-1 cleavage and activation of IL-1*β*. Further investigation is warranted to elucidate the molecular mechanism(s) of BRAT-mediated IL-1*β* expression since increased IL-1*β* expression may represent an early step contributing to OC.

## 1. Introduction

Ovarian cancer (OC), an inflammation associated cancer, is the deadliest gynecologic malignancy and is the 9th most common cancer among women [1]. Approximately 22,240 new cases are diagnosed and 14,030 women die of the disease annually [1]. Since most OC are diagnosed in late stage, the 5-year survival is approximately 27% [1].

Most OCs are epithelial ovarian carcinomas traditionally thought to arise from the ovarian surface epithelium (OSE) [2]; though more recently, studies have suggested that OC may arise from the fallopian tube epithelium [3, 4]. The etiology of the disease is not completely understood, but family history (FH) is the strongest risk factor for the development of epithelial OC [5]. Hereditary OCs are often associated with mutation of the tumor suppressor breast cancer susceptibility gene 1 (BRCA1) [6]. Carriers of the BRCA1 mutation have a 30% risk of developing OC during their lifetime [7]. BRCA1 plays a role in DNA damage response, cell cycle signaling, recruitment of chromatin modifying proteins, interaction with transcription factors, and ubiquitin ligase activity [8]. Loss of these functions may contribute to the development of cancer by promoting genomic instability and accumulation of cancer-causing mutations. Mutation of the BRCA1 gene can also result in either “loss of function” or “gain of function” with appearance of novel truncated protein products, respectively (reviewed in [9]). Among possible gain-of-function BRCA1 mutations, the 185delAG mutation is one of the most common founder mutations and is associated with a 66% lifetime risk of developing OC [10].

The 185delAG BRCA1 truncated mutant, BRAT, is the result of a deletion of two nucleotides in the second exon of the BRCA1 gene leading to a reading frame shift and a premature stop codon at position 39. Previously, we demonstrated that human OSE cells with the BRAT mutation exhibited enhanced apoptosis and caspase-3 activation [11] as well as diminished levels of phosphorylated Akt, cellular inhibitor of apoptosis 1 (cIAP1), and X-linked inhibitor of apoptosis protein (XIAP) [12]. We also found that BRAT-mediated maspin expression was correlated with enhanced chemosensitivity [13] which is in agreement with clinical reports of increased survival in patients with elevated maspin levels [14]. Microscopic examination of ovarian specimens obtained following prophylactic oophorectomy from women with FH of OC indicated that greater than 85% presented with two or more abnormal OSE histologic features such as surface epithelial pseudostratification, surface papillomatosis, cortical invaginations of OSE, epithelial inclusion cysts, and epithelial hyperplasia [15]. Likewise, overtly normal OSE from women with a FH of breast and/or OC (FHOSE) in culture show an increased autonomy of the epithelial phenotype in terms of expression of the epithelial differentiation marker CA125 [16], persistence of an epithelial morphology [16], and reduction in epithelial-mesenchymal conversion as noted by the maintenance of high keratin expression, but a reduction of collagen type II expression compared with no family history (NFH) OSE [17]. Since OSE become more committed to an epithelial phenotype in the course of carcinogenesis, these reports coupled with our previous findings suggest that preneoplastic characteristics may already exist in overtly normal OSE in some women with a strong FH of breast and ovarian cancer.

Inflammation of the ovarian epithelium has also long been associated with increased risk for OC [18–21]. By promoting a local pelvic inflammatory reaction, endometriosis has been associated with increased risk for endometrioid adenocarcinoma and clear cell carcinoma of the ovary [22–25]. Likewise, chronic pelvic inflammatory disease, often resulting from infection, also supports a role for inflammation and increased risk for OC [26, 27]. Lastly, epidemiological studies suggest that incessant ovulation causes rapid cycles of OSE division associated with repeated ovulatory traumatization and repair of the ovulatory defect [28]. Ultrastructural and histochemical studies of OSE* in situ* have shown that OSE migrates and proliferates extensively during repair of the OSE after ovulation [29, 30]. During ovulatory repair, OSE is exposed to proinflammatory mediators such as cytokines, chemokines, matrix-remodeling enzymes, and various growth factors that can result in an increased risk for malignant transformation [31, 32]. Reports of OC associated with ovarian hyperstimulation due to fertility drugs [33, 34] further support a role for inflammatory traumatization in OC.

Interleukin-1 beta (IL-1*β*) is a proinflammatory cytokine mainly produced by monocytes and macrophages [35], but which can also be produced by endothelial cells, fibroblasts, and epidermal cells in response to bacterial or innate immunity stimulation [36]. Interestingly, normal and malignant epithelial ovarian cells also produce IL-1*β* [2]. IL-1*β* is translated into a 31 kDa inactive precursor form that is cleaved intracellularly by caspase-1 into an active 17 kDa secreted form [35]. The aim of this study was to investigate the* in vitro* production of IL-1*β* in human OSE cell lines carrying the 185delAG BRCA1 mutation in order to determine whether enhanced IL-1*β* in these cells could contribute to an inflammatory phenotype.

## 2. Materials and Methods

### 2.1. Cell Culture and Transfection

The following SV 40-Large T-Antigen transfected human OSE (IOSE) cell lines were used: IOSE118 derived from a normal patient with a FH of breast and/or OC with wtBRCA1 status confirmed [13]; IOSE 121 derived from a normal patient with NFH of breast and/or OC though BRCA1 mutation status was not determined; and IOSE 3261-77 and IOSE 1816-686 derived from normal patients with a FH of breast and/or OC as well as confirmed carriers of the BRCA1 185delAG mutation. IOSE cells were cultured in Medium 199/MCDB 105 (Sigma, St. Louis, MO) with 10% fetal bovine serum (FBS) and gentamicin. Stable PCDNA (pcDNA3.1) and BRAT clones (BRATc1, BRATc2) were generated by transfection of 2.5 × 10^5^ IOSE 118 cells with 2.5 *μ*g of pcDNA3.1 or Flag-BRAT with G418 resistance gene as previously described [13] using Program X-005, Kit V, and the Nucleofector device (Amaxa/Lonza, Walkersville, MD). Stable cells were maintained in 1 mg/mL G418 selection media and BRAT clones were confirmed to express BRAT by RT-PCR [13]. All cells were incubated at 37°C with 5% CO_2_. For knockdown studies, cells were cotransfected with 1.5 *μ*g ON-TARGETplus siRNA (siCREB, siCON) from Dharmacon (Chicago, IL). For treatment with IL-1 receptor antagonist (IL-1Ra), stable BRAT clones were washed with phosphate buffered saline (PBS) and media containing 0.1% FBS were added to the cells along with varying concentrations of IL-1Ra (R&D Systems, Inc., Minneapolis, MN). After 6 hours, the media were removed and RNA was extracted from the cells for PCR analyses as described below.

### 2.2. Western Immunoblot

Cells were washed in PBS, trypsinized, pelleted, and washed 1-2 times in cold PBS. Cells were lysed for 30 minutes on ice in modified CHAPS buffer, and the lysate was centrifuged at 115,000 ×g, at 4°C for 1 hour. Then 30 *μ*g of protein was separated via 10% sodium dodecyl sulfate polyacrylamide gel electrophoresis (SDS-PAGE). Proteins were transferred to polyvinylidene fluoride (PVDF) membranes, dried, and blocked in 5% milk in Tween 20-Tris buffered saline. Blots were incubated in their respective primary antibodies overnight, followed by incubation with a horseradish peroxidase- (HRP-) conjugated secondary antibody (Fisher, Pittsburgh, PA), and developed via enhanced chemiluminescence substrate (ECL) (Pierce/Fisher, Pittsburgh, PA). Antibodies used were IL-1*β* (1 : 1000) Cat. # 2022 (Cell Signaling Technology, Beverly, MA), Actin clone AC-40 (1 : 10,000) Cat. # 4700 (Sigma, St. Louis, MO), Apoptosis-associated Speck-like protein containing a C-terminal caspase recruitment domain (ASC) (1 : 1000) Cat. # sc-271054 (Santa Cruz Biotechnology, Inc., Dallas, TX), Caspase-1 (1 : 1000) Cat. # 2225 (Cell Signaling Technology, Beverly, MA), CREB (1 : 1000) Cat. # 9104 (Cell Signaling Technology, Beverly, MA), phospho-CREB (1 : 1000) Cat. # 9196 (Cell Signaling Technology, Beverly, MA), NACHT, LRR, and PYD domains-containing protein 3 (NALP3) (1 : 1000) Cat. # sc-134306 (Santa Cruz Biotechnology, Inc., Dallas, TX). Western blot quantification was done by ImageJ software normalizing band strength to the respective actin band.

### 2.3. Enzyme-Linked Immunosorbant Assay

For conditioned media (CM) analyses, medium containing 0.1% FBS was added to cells 24 hours after transfection/plating. After 24 hours, cells were counted and CM were collected and centrifuged to remove debris, aliquoted, and stored at (−80°C). For the IL-1*β* Enzyme-Linked Immunosorbant Assay (ELISA), CM were also concentrated 17x using Amicon Ultra-15 Centrifugal Filter Unit with Ultracel-10 membrane (Millipore, Billerica, MA) before storage at (−80°C). To assess the presence of mature IL-1*β*, an ELISA (R&D Systems, Inc., Minneapolis, MN) was performed on concentrated CM samples in triplicate according to the manufacturer's protocol. Fluorescence was read on an ELx800 Absorbance Microplate Reader (Biotek, Winooski, Vermont) using Gen5 Data Analysis Software (Biotek, Winooski, Vermont). Resultant values were derived from a standard curve and expressed as the mean IL-1*β* concentration of triplicate samples ± standard error. When cell viability varied significantly, IL-1*β* concentration was normalized to average cell number at time of CM collection.

### 2.4. Polymerase Chain Reaction

RNA samples were isolated using TRIzol reagent from Invitrogen (Carlsbad, CA) as per the manufacturer's protocol and DNAse-treated.

For BRAT semiquantitative PCR to confirm transfection, 1 *μ*g total RNA, oligo(dT), and reverse transcriptase were used to generate single-strand cDNA as previously described [13]. The cDNA samples were amplified using the Applied Biosystems GeneAmp RNA PCR Core Kit (Foster City, CA). Primers used were Flag-BRAT sense (CGATGACAAAATGGATTTATCTGC), Flag-BRAT antisense (GAGACAGGTTCCTTCATCAACTCC), actin sense (GGGAATTCAAAACTGGAACGGTGAAGG), and actin antisense (GGAAGCTTATCAAAGTCCTCGGCCACA). The amplified products were separated by electrophoresis on a 10% polyacrylamide gel, stained with SYBR Green (Lonza, Rockland, ME), and photographed with the Kodak EDS 120 Digital Analysis System. The net intensity of each band was normalized to the respective endogenous control band.

For quantitative PCR, 100 ng total RNA was reverse-transcribed to generate single-strand cDNA as previously described [13]. The cDNA samples were amplified in triplicate using Fast SYBR Green Master Mix (Applied Biosystems) on an Applied Biosystems Step One Plus instrument. Primers used were IL-1*β* sense (TCCAGGGACAGGATATGGAG), IL-1*β* antisense (TCTTTCAACACGCAGGACAG), and actin (same as above). RQ (relative mean mRNA expression level) was calculated by the Step One software version 2.0. Using standard curves constructed for target and endogenous control genes, an arbitrary quantitative gene expression value was determined from the threshold cycle (Ct) for each gene for each sample. Target gene values were normalized to control gene values, and fold difference was determined by dividing by the designated reference/calibrator sample.

### 2.5. Dual-Luciferase Assay

Stable 118 pcDNA3.1 or BRAT clones were transfected with 0.15 *μ*g Renilla luciferase reporter and 1.5 *μ*g IL-1*β* luciferase promoter deletion constructs pIL1(4.0 kb)LUC (−4000), pIL1(3.1 kb)LUC (−3100), pIL1(1.8 kb)LUC (−1800), pIL1(0.9 kb)LUC (−900), and pIL1(0.5 kb)LUC (−500). Twenty-four hours later, cells were collected in Promega Passive Lysis Buffer and subjected to two freeze-thaw cycles. Lysates were centrifuged at 10,600 ×g for 1 minute at 4°C, and the supernatant was collected. Luciferase activity was assessed in triplicate using a manual luminometer and the Promega Dual Luciferase Assay System according to the manufacturer's protocol. For knockdown reporter studies, siRNA was cotransfected and cells were collected 48 hours after transfection.

### 2.6. Immunoprecipitation

Cells from stable 118 pcDNA3.1 and BRAT clones were washed with PBS and incubated on ice with RIPA lysis buffer containing protease inhibitors. After 15 minutes, the cells were removed by scraping, the lysates were incubated for 60 minutes on ice, and then the lysates were centrifuged at 10,000 ×g for 10 minutes at 4°C. Two micrograms of primary antibody was incubated with 1 mg of whole cell lysate for 2 hours at 4°C. Protein A/G agarose suspension was added to lysate and antibody mixture followed by incubation at 4°C on a rocker overnight. The suspension was collected by centrifugation at 500 ×g for 2 minutes at 4°C. The pellet was washed with RIPA buffer containing protease inhibitors followed by analysis by western immunoblotting.

### 2.7. Statistics

For real-time PCR, relative gene expression was expressed as the average fold change (RQ) of RQ_min_ and RQ_max_ calculated as 2^−(ΔΔCt+*T*×SD(ΔCt))^ and 2^−(ΔΔCt+*T*×SD(ΔCt))^, respectively, at the 95% confidence interval, for 5 degrees of freedom and where SD = standard deviation, *T* = confidence level and Ct = cycle threshold. For ELISA and reporter assays, Student's *t*-tests were performed to assess statistical difference between means of triplicates ± standard error from three separate experiments.

## 3. Results

### 3.1. IL-1*β* Levels Are Increased in IOSE Cells Carrying the BRCA1 185delAG Mutation

**Figure 1:**
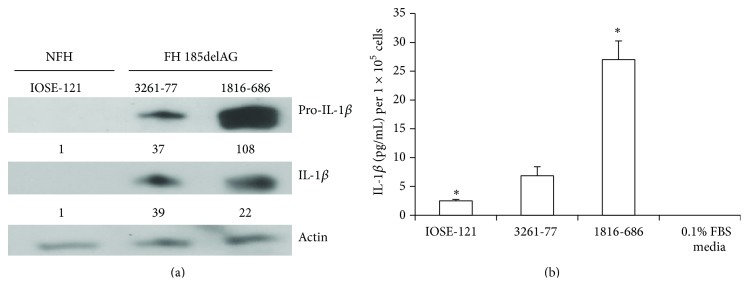
IL-1*β* protein levels are increased in 185delAG BRCA1 mutation carriers. (a) Normal NFH and FH BRCA1 185delAG IOSE cells were analyzed for precursor (pro-) and cleaved IL-1*β* protein expression via western blot. Blots were then stripped and probed for *β*-actin as a loading control. Values represent relative densitometry. (b) Cells were plated in triplicate at similar densities and conditioned media were collected as described. IL-1*β* ELISA activity assay was performed in triplicate and the results are expressed as the mean ± standard error. Symbol (*∗*) denotes statistical significance at the 0.04 confidence level.

To determine the relationship between the BRCA1 185delAG mutation and protein levels of IL-1*β*, we compared IL-1*β* protein levels in human OSE cell lines that endogenously carry this mutation to those with wild-type BRCA1. Both FH IOSE 3261-77 and 1816-686 cell lines with confirmed 185delAG mutation had 7-fold and ≥13-fold higher active IL-1*β* protein levels, respectively, as measured by western blot than the NFH cell line (IOSE-121) (Figure 1(a)). Likewise, 3261-77 and 1816-686 cell lines had 2- and 10-fold, respectively, higher levels of secreted IL-1*β* protein in their CM as measured by ELISA than the IOSE-121 cell line (Figure 1(b)).

### 3.2. BRAT Transfection Increases Levels of IL-1*β*

**Figure 2:**
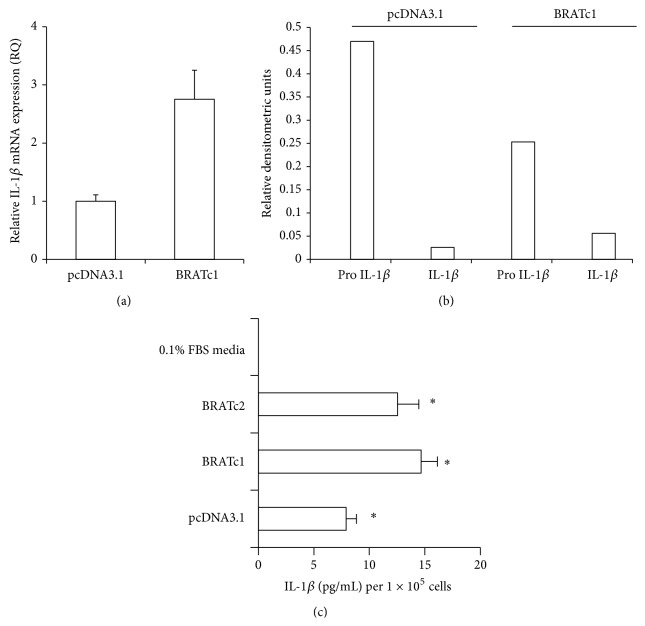
IL-1*β* protein and message levels are increased in stably transfected BRAT cells. (a) Cells stably expressing BRAT or the pcDNA3.1 cells were plated at equal densities and RNA was isolated, DNAse-treated, and reverse-transcribed. Real-time PCR was performed in triplicate for IL-1*β* and actin using SYBR Green detection and the data are expressed as the mean fold difference ± standard error. (b) pcDNA3.1 and BRATc1 cells were analyzed for precursor (pro-) and cleaved IL-1*β* protein expression via western blot. Blots were then stripped and probed for *β*-actin as a loading control. Values represent relative densitometry. (c) pcDNA3.1, BRATc1, and BRATc2 cells were plated in triplicate at similar densities and conditioned media were collected. IL-1*β* ELISA activity assay was performed in triplicate and the data are expressed as the mean ± standard error. Symbol (*∗*) denotes statistical significance at the 0.01 confidence level between BRAT and PCDNA3.1 cells.

To confirm that increased intracellular and secreted IL-1*β* levels are related to the presence of BRAT, we analyzed IOSE-118 cells stably transfected with PCDNA or Flag-BRAT for IL-1*β* message and protein levels. BRATc1 had approximately 3-fold higher levels of IL-1*β* RNA as measured by real-time PCR than the pcDNA3.1 cell line (Figure 2(a)). Furthermore, densitometric analyses indicated that 22% of cellular pro-IL-1*β* in BRAT cells was cleaved into its active form while only 5.5% of cellular pro-IL-1*β* in pcDNA3.1 cells was cleaved into its active form (Figure 2(b)). Lastly, BRATc1 and BRATc2 both had 2-fold and 1.5-fold, respectively, higher levels of secreted IL-1*β* protein levels as measured by ELISA than the pcDNA3.1 cells (Figure 2(c)).

### 3.3. BRAT-Mediated IL-1*β* Promoter Activity Is Partially Mediated by CREB

**Figure 3:**
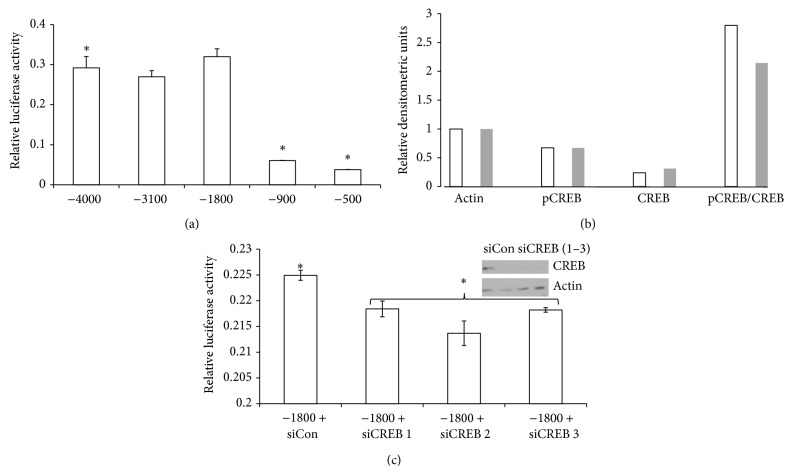
CREB sites within the IL-1*β* promoter partially mediate enhanced IL-1*β* mRNA expression in BRAT cells. (a) Cells stably expressing BRAT were transiently transfected with the indicated IL-1*β* deletion reporter constructs and a Renilla constitutive luciferase reporter plasmid for normalization. Lysates were collected, subjected to two freeze-thaw cycles, and assayed in triplicate on a manual luminometer using Promega's Dual Luciferase Assay Kit. Luciferase activity was normalized to Renilla luciferase activity for each triplicate, averaged, and results are expressed as mean reporter activity ± standard error. (b) Stable BRAT (white bars) and pcDNA3.1 (grey bars) cells were analyzed by western immunoblot for comparison of cellular levels of phospho-CREB, CREB, and actin. The results are expressed in relative densitometric units. (c) Cells stably expressing BRAT cells were cotransfected with nontargeting control siRNA (siCon) or siRNA targeting CREB, collected, and assayed similarly to (a). Protein lysates were collected in parallel for knockdown analyses and CREB protein silencing demonstrated by western blot (inset). Symbol (*∗*) denotes statistical significance ≤ 0.03 confidence level.

To determine which region of the *IL-1β* promoter was required for the BRAT-mediated* IL-1β* promoter activation, we utilized dual-reporter assays in BRAT cells using five truncated* IL-1β* promoter luciferase reporter plasmids. There was no difference in reporter activity between deletion constructs (−4000), (−3100), and (−1800) (Figure 3(a)). However, reporter activity was diminished by 80% between deletion constructs (−1800) and (−900) (Figure 3(a)) suggesting that the 900 bp region between the (−1800) construct and the (−900) construct contributes to BRAT-mediated* IL-1β* promoter activity.

To further evaluate BRAT-mediated* IL-1β* promoter activation, we performed an online transcription factor prediction analysis of the 900 bp promoter region identified earlier to determine potential transcription factor binding sites using the prediction software, PROMO [37, 38]. Our analyses identified two potential CREB binding sites within the 900 bp promoter region. Further, densitometric analyses of western immunoblots performed for phospho-CREB and CREB levels revealed 30% increased levels of phospho-CREB in BRAT cells (Figure 3(b)). Based on this observation, we used pooled siRNA to knockdown CREB and western blot to confirm CREB silencing. We found that CREB knockdown resulted in partial loss of* IL-1β* promoter activity using the (−1800) reporter plasmid (Figure 3(c)).

### 3.4. Caspase-1, ASC, and NALP3 Protein Levels Are Increased in BRAT Cells

**Figure 4:**
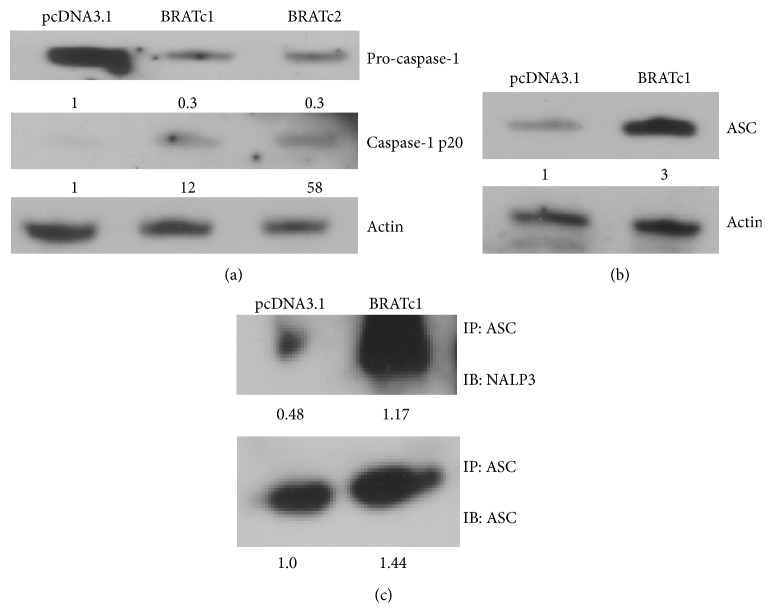
BRAT alters levels of inflammasome constituents. (a) Cells stably expressing pcDNA3.1 or BRAT (BRATc1 and BRATc2) were analyzed for precursor (pro-caspase-1) and cleaved caspase-1 (caspase-1 p20) protein levels via western blot. Blots were then stripped and probed for *β*-actin as a loading control. Values represent relative densitometric units. (b) Stable pcDNA3.1 or BRATc1 cells were analyzed for ASC protein expression via western blot. Blots were then stripped and probed for *β*-actin as a loading control. Values represent relative densitometric units. (c) Lysates obtained from stable pcDNA3.1 and BRATc1 cells were immunoprecipitated with ASC antibody and then analyzed via western blot for ASC and NALP3. Values represent densitometric units.

To confirm intracellular cleavage of the inactive IL-1*β* into its active form, we measured caspase-1 levels, the known intracellular activator of IL-1*β*. Stably transfected pcDNA3.1 cells had about 3-fold more inactive precursor caspase-1 protein levels than BRAT cells as seen by western blot (Figure 4(a)). However, stably transfected BRAT cells showed up to a 50-fold increase in active caspase-1 by western blot (Figure 4(a)). Given that BRAT might increase IL-1*β* activation by caspase-1-mediated cleavage, we sought to determine if the NALP3-ASC inflammasome, which cleaves pro-caspase-1 into its active form, was enhanced in BRAT cells. Western immunoblots confirmed a 3-fold increase of ASC protein levels in BRAT cells compared to the pcDNA3.1 cells (Figure 4(b)). Likewise, immunoprecipitation followed by densitometric analyses showed that, when normalized to cellular protein, both ASC and NALP3-bound ASC protein levels were 44% and 250% higher, respectively, in BRAT cells (Figure 4(c)).

### 3.5. IL-1Ra Suppresses Proinflammatory Mediators in BRAT Cells

**Figure 5:**
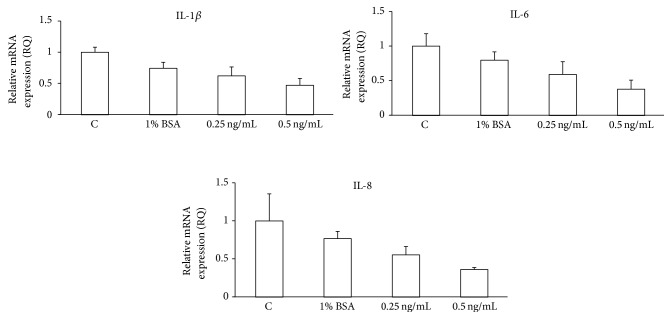
IL-1Ra inhibits IL-1*β*, IL-6, and IL-8 mRNA expression in BRAT cells. Cells stably expressing BRAT were plated at equal densities, treated with recombinant IL-1Ra, and collected. RNA was isolated, DNAse-treated, and reverse-transcribed. Real-time PCR was performed in triplicate from two separate experiments for IL-1*β*, IL-6, IL-8, and actin using SYBR Green detection. Data are expressed as the mean fold change in gene expression ± standard error.

To evaluate the potential autofeedback loop of IL-1*β* on OSE cells, we treated stably transfected BRAT-containing IOSE cells with the IL-1 receptor antagonist, IL-1Ra. Then, we measured mRNA expression levels of known IL-1*β* downstream transcription targets: IL-1*β*, IL-6, and IL-8 by real-time PCR. Treatment of BRAT cells with IL-1Ra resulted in up to a 50% loss of mRNA expression levels of IL-1*β*, IL-6, and IL-8 (Figure 5).

## 4. Discussion

The results of the present study show that the 185delAG BRCA1 mutant protein, BRAt, increases IL-1*β* mRNA and protein levels in immortalized human OSE cell lines. Interestingly, compared to controls, increased levels of IL-1*β* varied among IOSE cell lines and may reflect differences in BRCA1 185delAG penetrance, BRAt expression, and/or wtBRCA1 expression [11, 13]. Therefore, it is important to note that inherent differences of wtBrca1 expression among cell lines derived from patients endogenously carrying the 185delAG mutation can limit understanding the independent functions of BRAt in these cells. For these reasons, we transitioned our studies into transiently or stably transfected 185delAG cell lines in a confirmed wtBRCA1 background.

We also demonstrated that BRAT-dependent expression is transcriptionally mediated, in part, via CREB binding sites. In addition, we found that BRAT-dependent activation of IL-1*β* protein by caspase-1 may be enhanced by increased expression of the NALP3-ASC inflammasome. While further investigation is required to discover the exact mechanism of enhanced NALP3-ASC protein expression in BRAT cells, we suspect that ASC expression may be CREB-dependent or AP1-dependent since the current and prior studies indicate BRAT-mediated gene expression through CREB and AP1 sites [39]. Further, CREB binding sites are likely present in the ASC promoter according to commercially available promoter ChIP assay (Qiagen, Valencia, CA) program analysis. Lastly, we confirmed that IL-1*β* plays a role in expression of proinflammatory mediators, IL-6 and IL-8, via the IL-1 receptor suggesting that BRAT might stimulate a proinflammatory environment that could promote OC oncogenesis.

Establishing animal and cell models of OC oncogenesis has proven to be difficult. Spontaneous development of OC in animal models occurs with a low frequency and highly variable phenotypes [40]. Furthermore, OC lacks a clear molecular profile making it difficult to elucidate a universal cause in OC initiation or progression [41–43]. Loss of some or all wild type functions of the BRCA1 gene product due to gene mutation is commonly associated with enhanced breast cancer and OC risk (reviewed in [9]).* BRCA1* gene mutations resulting in a premature stop codon are generally susceptible to nonsense-mediated messenger RNA (mRNA) decay. However, two common risk-associated BRCA1 mutations, 185delAG and 5382InsC, were found to be unaffected by mRNA decay [44, 45]. This study utilizes a stable OSE cell line model with intact endogenous biallele* BRCA1* along with the 185delAG BRCA1 mutant transcript. By retaining a BRCA1 wild type background, we are able to conclude that changes observed are due to the presence of the 185delAG BRCA1 mutation and not due to the partial or complete loss of BRCA1. This is in keeping with previous studies that have shown other BRCA1 mutations with independent and novel gain-of-function roles in OC proliferation, chemosensitivity, tumorigenesis, and apoptosis [46, 47].

Inflammation has long been suggested to contribute to tumor initiation, promotion, and progression [48]. Many components of the inflammatory pathway, including free radicals, cytokines, cyclooxygenase-2 (COX-2), inducible nitric oxide synthase (iNOS), and vascular endothelial growth factor (VEGF) have been implicated in the development of various malignancies, including OC [49]. Immunohistochemical analyses revealed increased COX-2 expression in nonmucinous ovarian tumors and increased COX-2 expression correlating with poor prognosis [50]. Likewise, the present results are in keeping with others who reported a relation between proinflammatory mediators and increased OC risk. A case-control study by Clendenen et al. confirmed a positive association between circulatory inflammatory cytokines, IL-2, IL-4, IL-6, IL-12, and IL-13, and OC risk [51]. Also, increased serum levels of the inflammatory marker CRP have been associated with an increased risk of OC [20, 52]. Yellapa et al. demonstrated increased expression of the proinflammatory cytokine, IL-16, in serum and tissue of early and late stage OC patients as well as increased IL-16 serum levels during the progression from normal to early and late stage OC progression in hens [53]. Further, since increasing IL-16 serum levels preceded detection of OC by transvaginal ultrasound, it is tempting to speculate that monitoring proinflammatory mediators, such as IL-1*β*, may signal disease in women at high risk for OC before detection by conventional means.

Epidemiological studies suggest that long-term usage of nonsteroidal anti-inflammatory drugs (NSAIDs) is associated with decreased OC risk [54–56]. Further,* in vitro* and* in vivo* use of phytochemicals [57] exert anti-inflammatory activity related to reduced OC progression by stabilizing p53 [58], enhancing cisplatin sensitivity [59], inhibiting cell growth and VEGF expression [60], and suppressing IL-6 function [61]. There are currently three IL-1 inhibitors used in the treatment of nonmalignant inflammatory conditions: recombinant IL-1Ra, anakinra; IL-1 trap, rilonacept; and monoclonal anti-IL-1*β* antibody, canakinumab [62]. Consequently, premalignant targeting of the proinflammatory mediator IL-1*β* and its upstream processors, caspase-1 and/or the NALP3 inflammasome, specifically in 185delAG BRCA1 mutation carriers, may reduce the risk of malignant transformation initiated by chronic inflammation.

Lastly, tumors can develop within and in response to their inflammatory microenvironment. For example, Kim et al. observed localized IL-1 expression among normal, benign, and malignant canine mammary tumors such that IL-1 could not be detected in normal tissue while concentrated IL-1 expression was noted in the stroma of benign tumors and diffuse expression in malignant and metastatic tumors [63]. Similarly, IL-1*β* secreted by OC cells suppresses p53 expression in cancer-associated fibroblasts [64]. In OC, IL-1*β* also promotes invasiveness and tumor angiogenesis and induces immune suppression [65]. Constitutive production of IL-1*β* by human ovarian carcinoma cell lines [66] enhances their invasion capacities by increasing expression of matrix metalloproteinase-1 and stimulating production of proangiogenic factors [52, 67]. Watanabe et al. showed that IL-1*β* produced by OC cells induced mesothelial cell beta1-integrin-dependent peritoneal metastasis [68]. Increased levels of IL-1*β* found in the serum and/or the ascites of OC patients have been associated with decreased survival [69]. Likewise, IL-1*β* can effect OC progression by altering the expression of other proinflammatory cytokines, such as IL-6 which is a downstream target of IL-1*β*. IL-6 is known to play a major role in OC progression and prognosis and may also be a potential marker of immunological and metabolic changes in OC [70]. Elevated levels of IL-6 have been found in the serum and ascites of OC patients [71] and are also associated with poor prognosis [2]. In support of the data reported herein, we recently found that urinary levels of IL-1*β* were generally 5 times greater in women with OC compared to healthy controls [72]. Interestingly, while exact BRCA1 mutational status could not be determined, we also found that urinary levels of IL-1*β* were 3 times higher among women with benign ovarian disease and a FH of breast/ovarian cancer compared with women with benign ovarian disease and lacking a FH of breast/ovarian cancer. Likewise, OC patients with a confirmed FH of cancer had urinary IL-1*β* levels several fold higher than OC patients without a known FH of cancer. Therefore, chronic presence of IL-1*β* within the ovarian microenvironment may enhance malignant transformation and underscores the complexity among cancer cells within their microenvironment for tumor progression.

## 5. Conclusions

Our data identify the proinflammatory mediator, IL-1*β*, as a novel target in OSE cells expressing the 185delAG BRCA1 mutation. Further, the mechanism by which BRAT regulates IL-1*β* expression is twofold: (1) CREB-dependent transcriptional control and (2) caspase-1 cleavage of pro-IL-1*β*. It is interesting to speculate that increases in IL-1B might be limited to 185delAG rather than common to multiple BRCA1 mutations. While we have not been able to study other BRCA1 mutations in similar detail, a recent study by Rebbeck et al. [73] suggests that differences in risk for breast and/or ovarian cancer may be linked to the locations of mutations in BRCA1 and BRCA2. Further, in prior publications, we have shown that targets downstream of 185delAG, including maspin, are unaffected when 185delAG is itself mutated to generate a further BRCA1 mutation [39] suggesting that the 185delAG BRCA1 mutation may promote tumorigenesis by a unique mechanism. Clearly, additional studies on the role of BRCA1 mutations to promote a chronic inflammatory phenotype are warranted since the potential also exists for IL-1*β* to serve as a predictor of OC and/or as a therapeutic target.
